# Spontaneous Forearm Hemorrhage in a Patient With Polycystic Kidney Disease: A Case Report

**DOI:** 10.7759/cureus.81864

**Published:** 2025-04-08

**Authors:** Tomohiro Nakajima, Akihiro Tabata

**Affiliations:** 1 Cardiovascular Surgery, Sapporo Medical University, Sapporo, JPN; 2 Cardiovascular Surgery, Kobayashi Hospital, Kitami, JPN

**Keywords:** cerebral bleeding, forearm bleeding, hemodialysis, polycystic kidney, renal failure

## Abstract

The patient is a 73-year-old woman. She had a brain hemorrhage at 53 years of age and underwent craniotomy and hematoma removal. At 55 years of age, she was treated for multiple renal cysts. An infection has spread to the existing renal cyst. The patient’s renal function gradually deteriorated, and she underwent hemodialysis at 56 years of age. At 58 years of age, an internal shunt was created using an autologous blood vessel in the left elbow fossa. Thereafter, she was hospitalized six times because of a cyst infection and received antibiotic treatment. At 72 years of age, she experienced a brain hemorrhage and received conservative treatment at our hospital. Presently, she presented with swelling in the left forearm. Upon further examination, bleeding was found in the left forearm muscles. Hemostatic surgery was performed under general anesthesia. Here, we report a rare case of forearm hemorrhage in a patient with comorbid cystic kidney disease.

## Introduction

Polycystic kidney disease (PKD) is among the most common inherited kidney disorders, with a reported prevalence of 1 in 400-1,000 individuals [1]. It is characterized by the progressive development of multiple renal cysts, leading to kidney enlargement and gradual loss of renal function. Its clinical manifestations include hypertension, hematuria, flank pain, and renal insufficiency. As the disease progresses, many patients eventually develop end-stage renal disease (ESRD) and require renal replacement therapy, such as hemodialysis or kidney transplantation; approximately 50% of patients with autosomal dominant PKD (ADPKD) develop ESRD by 60 years of age [2]. PKD is associated with an increased risk of bleeding due to vascular fragility and hypertension [3]. There are several reports of intracranial aneurysms, subarachnoid hemorrhage, and spontaneous bleeding events, such as intracystic hemorrhage and gross hematuria [4]. However, spontaneous bleeding from an arteriovenous (AV) fistula on the forearm is extremely rare in patients with PKD. Here, we report a rare case of active bleeding from an AV shunt arm that required surgical hemostasis in a patient with PKD.

## Case presentation

**Figure 1:**
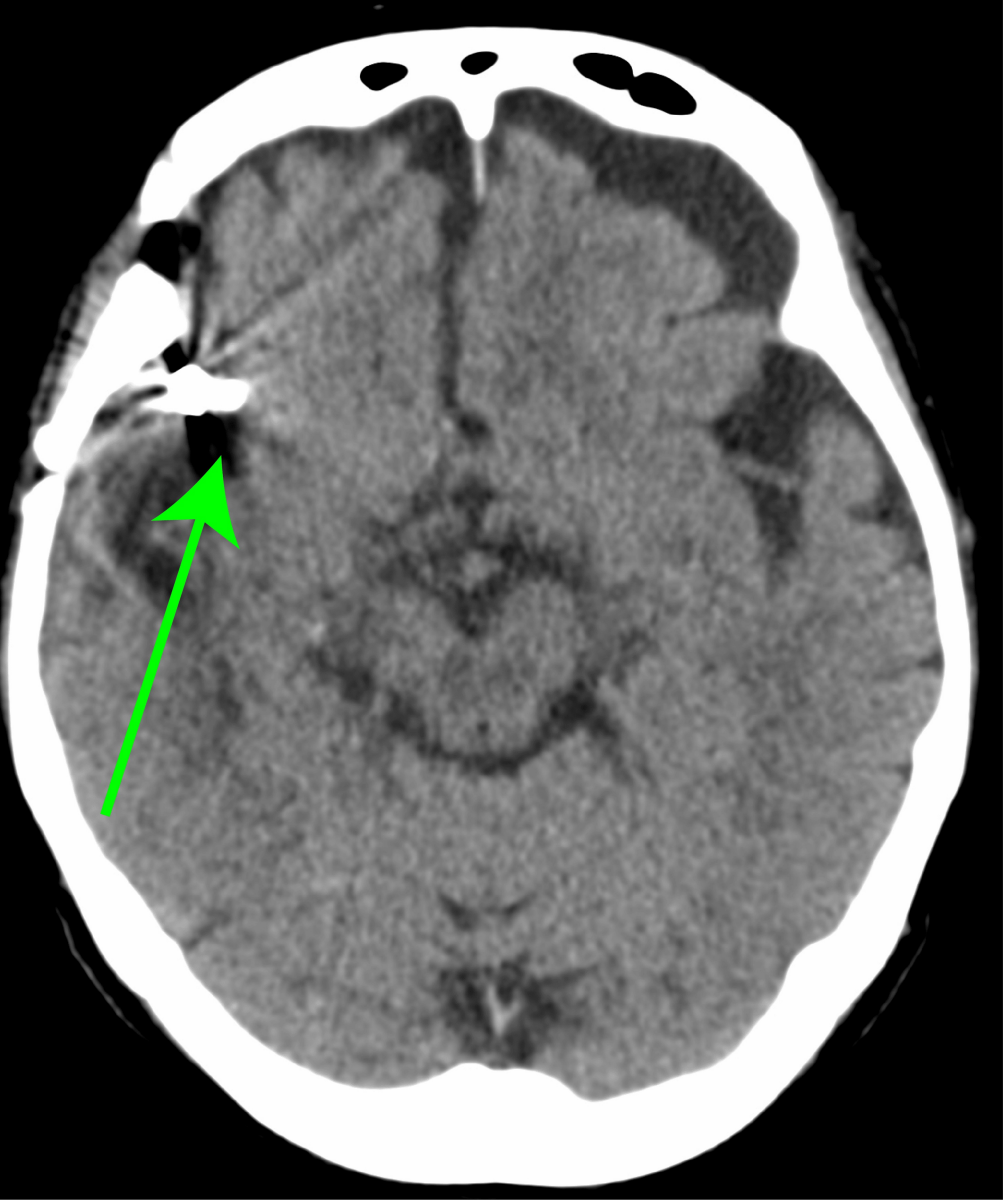
Brain computed tomography at the age of 53 years old. A clipping is observed in the right temporal lobe (green arrow).

The patient is a 73-year-old woman. She had a subarachnoid hemorrhage, at 53 years of age, and underwent craniotomy and hematoma removal surgery (Figure 1).

**Figure 2:**
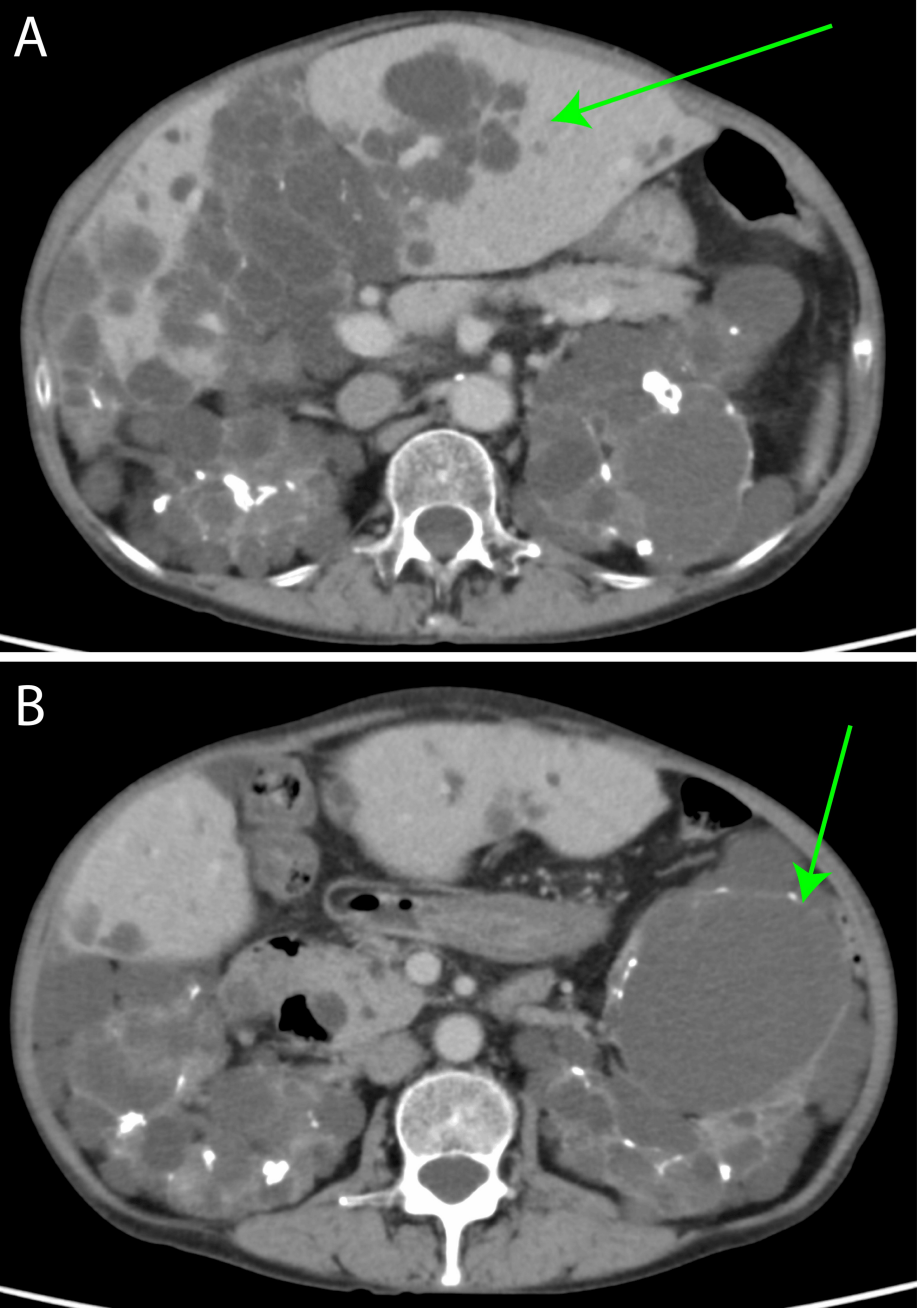
Contrast-enhanced computed tomography at the age of 55 years old. (A) Multiple cysts are observed in the liver and upper pole of the kidney (green arrow). (B) Numerous cysts are observed in the middle part of the kidney (green arrow).

At 55 years of age, she was treated for multiple renal cysts, which had been detected following an infection (Figure 2).

**Figure 3:**
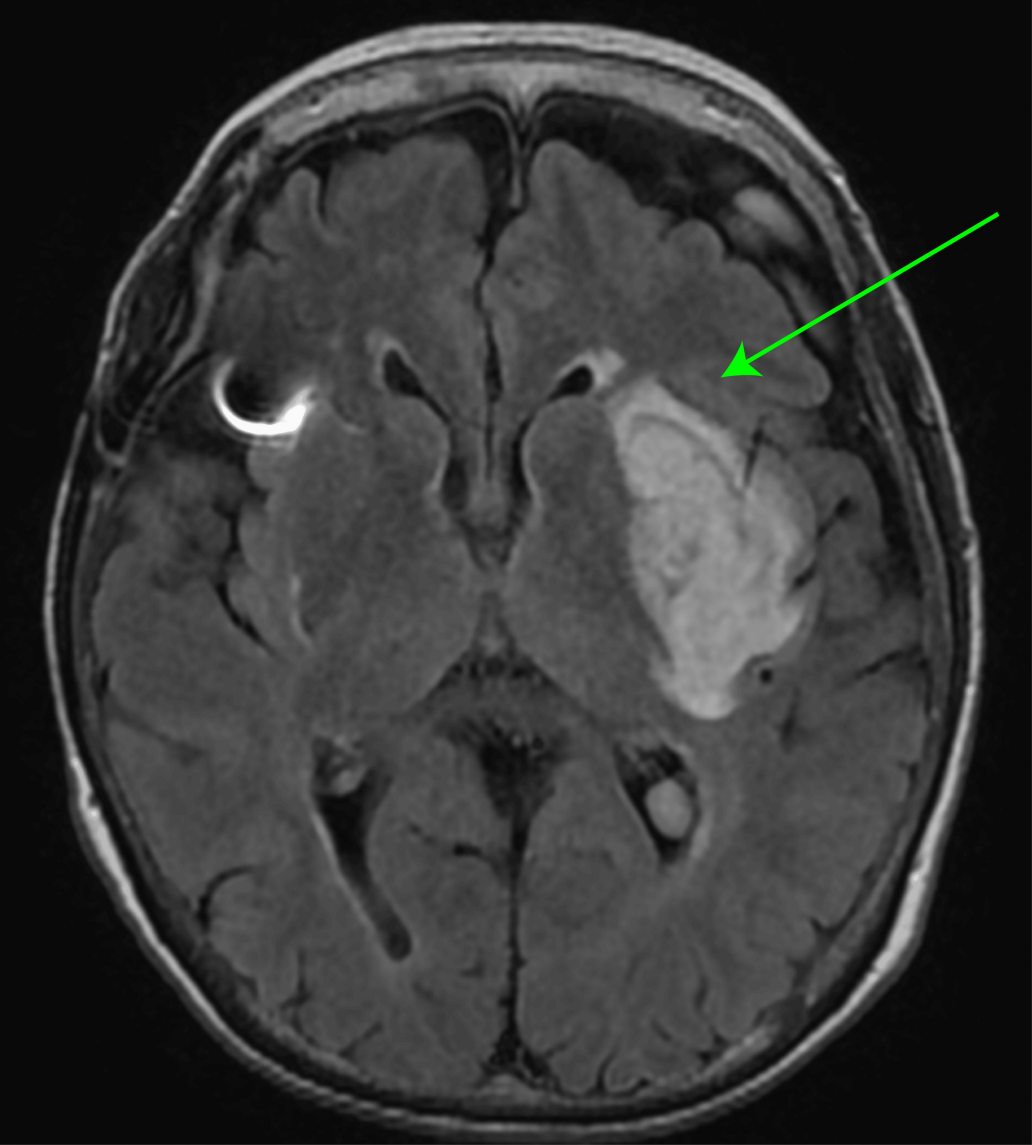
Magnetic resonance image at the age of 72 years old. Magnetic resonance imaging reveals a high-intensity lesion in the left thalamus, indicating a brain hemorrhage (green arrow).

The patient’s renal function gradually deteriorated, and she started undergoing hemodialysis at 56 years of age. An internal shunt was created using autologous blood vessels in the left forearm. At 58 years of age, an internal shunt was created using an autologous blood vessel in the left elbow fossa. Thereafter, she was hospitalized six times because of a cyst infection and received antibiotic treatment. At 72 years of age, she experienced a cerebral hemorrhage and received conservative treatment at our hospital (Figure 3).

**Figure 4:**
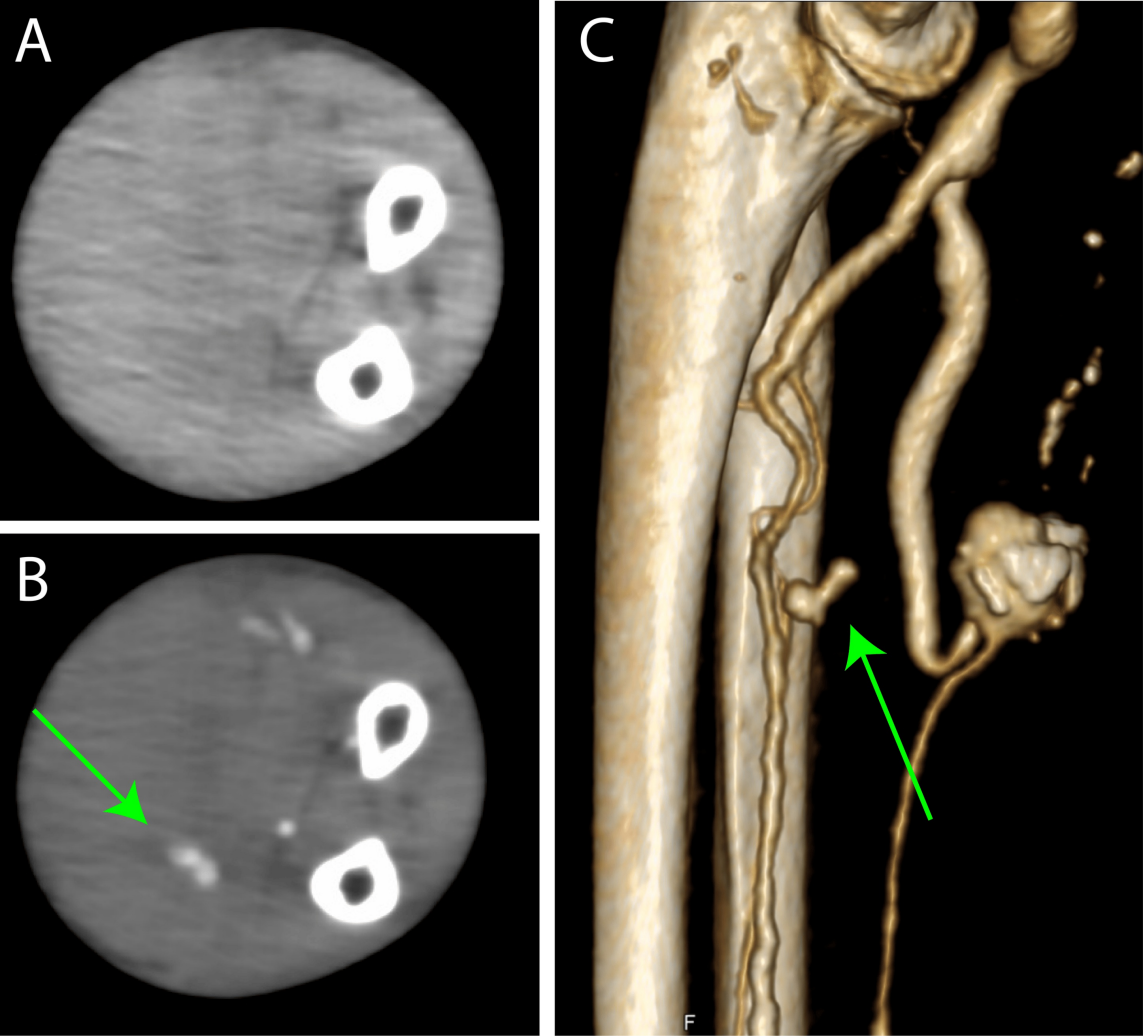
Preoperative images at the age of 72 years old. A computed tomography (CT) image of the left forearm. (A) Plain CT: Swelling of the left forearm is observed. (B) Venous phase: Venous bleeding is observed (green arrow). (C) Volume rendering image: Venous bleeding is observed (green arrow).

Recently, she presented with swelling in the left forearm. Upon further examination, bleeding into the left forearm muscle was found. Contrast-enhanced computed tomography (CT) revealed swelling in the left arm and rupture of the muscle branches (Figure 4). Because it was a difficult area to stop the bleeding, and the swelling of the left hand progressed, we had no choice but to consider surgery.

Under general anesthesia, a 10 cm skin incision was made on the left forearm, and hemostatic surgery was performed. The vein that flowed into the muscle was partially ruptured, and the site was clipped to stop the bleeding.

A drain was placed, the skin was closed, and surgery was completed. Thereafter, the bleeding subsided, and the swelling in the left forearm improved.

## Discussion

Here, we report a rare case of unexpected venous bleeding from a left forearm hemodialysis shunt in a patient with PKD. Although access to hemodialysis is crucial for patients with PKD progressing to ESRD, reports of shunt-related complications, particularly venous bleeding, are scarce. In this instance, a patient with a prolonged history of PKD-induced renal failure underwent left forearm AV fistula creation and subsequently experienced spontaneous venous bleeding, without any evident trauma or infection.

PKD is a systemic disease that causes cyst formation in the kidneys and other organs [5]. Vascular wall fragility is a common characteristic of patients with PKD, increasing the risk of bleeding complications at various locations, including cerebral aneurysms, abdominal aortic aneurysms, and gastrointestinal cystic bleeding [6]. Therefore, venous bleeding from hemodialysis shunts may be attributable to PKD-related vascular wall fragility. Furthermore, renal dysfunction in PKD can lead to coagulopathy. Although the patient’s prothrombin time-international normalized ratio (PT-INR) is within normal limits, other coagulation abnormalities, such as platelet dysfunction, are included [7]. Additionally, PKD can be associated with vascular anatomical variations; although no gross abnormalities were observed, subtle variations may have played a role [8].

Provided the difficulty in achieving hemostasis, surgical intervention is necessary. Although the literature on managing such bleeding in patients with PKD is limited, surgical hemostasis appears to be a viable option. This case highlights the importance of considering the unique characteristics of patients with PKD in hemodialysis access management. Further research is necessary to identify the risk factors and establish optimal treatment strategies for this rare complication. Future studies are needed to further investigate the risk factors of hemodialysis shunt bleeding in patients with PKD, and clinical studies are needed to determine the best treatment plans for this complication.

## Conclusions

Although access to hemodialysis is essential for patients with PKD and ESRD, bleeding from the shunt limb is uncommon. PKD is a systemic disease that predisposes patients to bleeding complications through various mechanisms, including vascular fragility and coagulopathy. Surgical hemostasis is a viable treatment option for intractable bleeding. This report emphasizes the importance of considering the unique characteristics of patients with PKD in hemodialysis access management and provides valuable insights for the clinical management of similar cases.
